# Prediction of response to medical therapy by serum soluble (pro)renin receptor levels in Graves’ disease

**DOI:** 10.1371/journal.pone.0195464

**Published:** 2018-04-05

**Authors:** Yuki Mizuguchi, Satoshi Morimoto, Shihori Kimura, Noriyoshi Takano, Kaoru Yamashita, Yasufumi Seki, Kanako Bokuda, Midori Yatabe, Junichi Yatabe, Daisuke Watanabe, Takashi Ando, Atsuhiro Ichihara

**Affiliations:** Department of Medicine II, Endocrinology and Hypertension, Tokyo Women’s Medical University, Tokyo, Japan; Hokkaido Daigaku, JAPAN

## Abstract

Antithyroid drugs are generally selected as the first-line treatment for Graves’ Disease (GD); however, the existence of patients showing resistance or severe side effects to these drugs is an important issue to be solved. The (pro)renin receptor [(P)RR] is a multi-functional protein that activates the tissue renin-angiotensin system and is an essential constituent of vacuolar H^+^-ATPase, necessary for the autophagy-lysosome pathway. (P)RR is cleaved to soluble (s)(P)RR, which reflects the status of (P)RR expression. In this retrospective study, we aimed to investigate whether serum s(P)RR concentration can be used as a biomarker to predict the outcome of antithyroid drug treatment in GD patients. Serum s(P)RR levels were measured in 54 untreated GD patients and 47 control participants. Effects of medical treatment with antithyroid drugs on these levels were investigated in GD patients. Serum s(P)RR levels were significantly higher in patients with Graves’ disease than in control subjects (P<0.005) and were significantly reduced after medical treatment for Graves’ disease. High serum s(P)RR levels were associated with resistance to antithyroid drug treatment, suggesting that serum s(P)RR concentration can be used as a useful biomarker to predict the outcome of antithyroid drug treatment in these patients. Patients with Graves’ disease with low body mass index showed higher levels of serum soluble (pro)renin receptor levels than those with high body mass index. In addition, in patients with Graves’ disease, serum triglyceride levels were negatively correlated with serum soluble (pro)renin receptor levels. All these data indicated an association between low nutrient condition due to hyperthyroidism and increased (pro)renin receptor expression in these patients, suggesting that (pro)renin receptor expression could be increased in the process of stimulating intracellular energy production via activating autophagy function to compensate energy loss.

## Introduction

Prorenin, an inactivated precursor of renin, is activated upon binding to the (pro)renin receptor [(P)RR] and can carry out the conversion of angiotensinogen to angiotensin I like renin [1]. Thus, the (P)RR plays an important role in regulating the tissue renin-angiotensin system (RAS) [1]. The (P)RR also triggers its own intracellular signaling transduction that activates the angiotensin (Ang) II-independent pathways, such as mitogen-activated protein kinase (MAPK) /extracellular signal-regulated kinase 1/2 (ERK1/2) and p38 [2]. Moreover, the (P)RR was previously identified as an accessory protein of vacuolar H^+^-ATPase (V-ATPase), which is an ATP-dependent proton pump that plays an important role in transportation of protons across plasma membranes and acidifies intracellular compartments [3] [4] [5]. (P)RR, which consists of 350 amino acids with a single transmembrane domain, is cleaved by furin to generate soluble (s)(P)RR, which is secreted into the extracellular space and found in blood [6]. These findings suggest that s(P)RR can serve as a biomarker reflecting the tissue RAS status [7–9].

Graves’ disease (GD) is an organ-specific autoimmune disease characterized as overproduction of thyroid hormones resulting from the stimulation of circulating thyroid-stimulating hormone (TSH) receptor antibodies (TRAb) [10]. GD has adverse effects on quality of life and is associated with an increased risk of death [11]. Current therapeutic options for GD include antithyroid drugs, radioactive iodine, and thyroidectomy [12]. Antithyroid drugs inhibit thyroperoxidase, which oxidizes iodide ions to form iodine atoms for addition onto tyrosine residues on thyroglobulin for the production of thyroxine or triiodothyronine [13]. These drugs have many advantages, including normalizing thyroid function in a short time, rarely causing hypothyroidism, and ameliorating immune disorder while avoiding radiation exposure and invasive procedures. For these reasons, it is generally well accepted as the first-line treatment for GD [14, 15]. However, the existence of patients showing resistance or severe side effects to these drugs is an important issue to be solved [16] [17] [18]. Therefore, it is ideal to predict the responsiveness of antithyroid drugs in selecting treatment option in these patients.

Several studies have implicated the RAS in other cardiovascular effects of thyroid hormones besides their direct effects on heart and vascular systems [19], suggesting that enhanced metabolism caused by thyroid hormones might control the production of the (P)RR and s(P)RR. In addition, a recent study demonstrated the direct molecular binding between (P)RR and pyruvate dehydrogenase complex [20], indicating that (P)RR may contribute to the aerobic glucose metabolism together with oxidative stress. This suggests that (P)RR expression might reflect the activity of oxidative phosphorylation enzyme including thyroperoxidase, and thus, blood s(P)RR concentration might predict the responsiveness of antithyroid drugs.

In the present study, we measured serum s(P)RR concentration to investigate its characteristics and to assess whether it can be used as a biomarker to predict the outcome of antithyroid drug treatment in these patients.

## Subjects and methods

### Study participants

Patients with untreated GD were age-matched with control subjects who had nonfunctional adrenal tumors, yet whose thyroid function was normal. All study participants visited our outpatient clinic from April 2014 to October 2015. Pregnant women and patients with essential hypertension (EH), diabetes mellitus, chronic kidney disease, or malignant disease were excluded. The study protocol was approved by the Ethics Committee of Tokyo Women’s Medical University (2303-R5) and registered in the UMIN Clinical Trial Registry (UMIN000006222), and all patients provided written informed consent.

### Blood pressure and pulse rate

Blood pressure and pulse rate were measured at our outpatient clinic in a sitting position at heart level after at least 5 minutes of rest. The first readings at each visit were used for this study.

### Blood examinations

For a period ranging from 6 to 18 months, blood samples were taken from study participants in the early morning after at least 15 minutes of sitting, both before and after treatment with antithyroid drugs (thiamazole or propylthiouracil). Blood sugar, hemoglobin A1c (HbA1c), blood urea nitrogen (BUN), creatinine, low density lipoprotein-cholesterol (LDL-C), high density lipoprotein-cholesterol (HDL-C), triglyceride (TG), TSH, free thyroxine (FT4), free triiodothyronine (FT3), and anti TRAb were measured by standard methods at our hospital. Serum levels of s(P)RR were measured using an ELISA kit (Takara Bio Inc, Otsu City, Japan) which consists of a solid-phase sandwich ELISA with antibody highly specific for s(P)RR [21]. The estimated glomerular filtration rate (eGFR) was calculated using the following equation:

$$eGFR\;(ml/min/1.73\;m^{2})=194\;x\;{creatinine}^{-1.094}\;x\;{age}^{-0.287}(x\;0.739\;if\;female)$$

[22]

### Study protocol

Serum s(P)RR levels were compared between untreated GD patients and control participants. Effects of medical treatment with thiamazole, an antithyroid drug, on serum s(P)RR levels were retrospectively investigated in GD patients for at least 6 months. Patients who revealed euthyroidism (showing normalization of both FT4 and TSH) or hypothyroidism after 6 months of treatment were defined as responders (R) and those who remained hyperthyroid were defined as non-responders (NR). Then, the association between serum s(P)RR levels before treatment and efficacy of the medical treatment was investigated. In addition, the association between body mass index (BMI) and serum s(P)RR levels was assessed in both GD patients and control participants. Lastly, the association of background factors such as age, body mass index, blood pressure, and laboratory results with serum s(P)RR levels in GD patients was explored.

### Statistical analysis

Results were expressed as mean ± S.D. Wilcoxon rank sum test and χ^2^-test were applied to compare two groups. Serum s(P)RR levels in GD patients before and after medical treatment were compared using a paired Student’s *t*-test. Associations between background factors and serum s(P)RR levels in GD patients were assessed using Spearman’s rank correlation analysis. Linear multiple regression analyses were used to identify possible determinants. All statistical analyses were performed using GraphPad Prism (Version 6) statistical program. The level of significance was defined as *P*<0.05.

## Results

### Comparisons of the basic characteristics between control participants and GD patients

Fifty-four patients with Graves’ disease and 47 control participants were enrolled in this study. Baseline characteristics of the study participants are shown in Table 1. There were no differences in gender, age, BMI, BP, and serum TG levels between normal participants and GD patients. Serum levels of LDL-C, HDL-C, and creatinine were significantly lower and HbA1c, eGFR, and serum levels of FT4, FT3 and s(P)RR were significantly higher in GD patients than in control participants.

**Table 1:** Characteristics of the study subjects.

	Con	GD	p
Gender (Male/Female)	10/37	13/36	0.157
Age (y.o.)	50 ± 4	48 ± 3	0.767
Body mass index (kg/m^2^)	20.3 ± 0.7	21.3 ± 1.1	0.344
Blood pressure			
Systolic blood pressure (mmHg)	122 ± 3	117 ± 3	0.311
Diastolic blood pressure (mmHg)	73 ± 3	68 ± 2	0.111
Blood tests			
Hemoglobin A1c (%)	5.27 ± 0.13	5.57 ± 0.09	0.007
LDL-cholesterol (mg/dl)	108± 6	69± 6	<0.001
HDL-cholesterol (mg/dl)	76 ± 4	57 ± 6	0.019
Triglyceride (mg/dl)	81 ± 9	81 ± 7	0.084
Creatinine (mg/dl)	0.69 ± 0.23	0.51 ± 0.03	<0.001
eGFR (ml/min/1.73 m^2^)	80.8 ± 4.17	124.2 ± 12.2	<0.001
FT4 (ng/dl) (reference range: 0.94–1.60)	1.19 ± 0.04	4.97 ± 0.36	<0.001
FT3 (pg/ml) (reference range: 2.40–4.00)	2.80 ± 0.11	17.27 ± 1.63	<0.001
TRAb (IU/l)	n.a.	25.58 ± 5.57	n.a.
s(P)RR (ng/ml)	19.70 ± 3.05	28.08 ± 5.29	<0.001

Con, Control; GD, Graves’ disease; eGFR, estimated glomerular filtration rate; TSH, thyroid stimulating hormone; FT4, free thyroxine; FT3, free triiodothyronine; TRAb, anti-TSH receptor antibody; s(P)RR, soluble (pro)renin receptor

### Effects of antithyroid treatment for GD on serum s(P)RR levels

Serum levels of FT4 (1.05 ± 0.11 ng/dl, P<0.001 compared with before treatment) and FT3 (2.68 ± 0.15 pg/ml, P<0.001) were significantly decreased and those of TSH were significantly increased (2.01 ± 0.36 μIU/l, P<0.001) by the antithyroid treatment for the patients with GD. However, 18 patients (58%) were classified as R (Responder) and 13 patients (42%) were classified as NR (Non-Responder). Serum s(P)RR levels were significantly reduced by the treatment (from 27.74 ± 5.83 ng/ml to 22.47 ± 2.67 ng/ml, P<0.005). Patients with higher (≥ 27 ng/ml) and lower (< 27 ng/ml) levels of serum s(P)RR before treatment showed significantly lower (10/21) and higher (8/10) rates after treatment, respectively, demonstrating responsiveness to the antithyroid treatment (relative risk, 2.63) (Table 2).

**Table 2:** Responses to antithyroid drugs and serum s(P)RR in patients with Graves’ disease.

	NR group	R group	Total
Serum s(P)RR levels ≥ 27 ng/ml	11	10	21
Serum s(P)RR levels < 27 ng/ml	2	8	10
	13	18	31
	RR: 2.63		

s(P)RR, soluble (pro)renin receptor; NR, non-responder; R, responder; RR, Relative Risk

While serum s(P)RR levels were significantly higher in the NR group than in the R group, there were no significant differences in other background factors including thyroid functions between NR and R groups (Table 3). These data suggested that serum s(P)RR concentration could be a biomarker to predict the responsiveness to the antithyroid treatment.

**Table 3:** Comparisons background factors between non-responder and responder groups.

	NR group (n = 13)	R group (18)	p
Gender (Male/Female)	8/5	14/4	0.157
Age (y.o.)	48 ± 4	49 ± 4	0.983
Body mass index (kg/m^2^)	21.6 ± 6.1	21.7 ± 4.6	0.776
Blood pressure			
Systolic blood pressure (mmHg)	119 ± 4	116 ± 5	0.805
Diastolic blood pressure (mmHg)	66 ± 3	68 ± 4	0.729
Blood tests			
Hemoglobin A1c (%)	5.74 ± 0.41	6.36 ± 0.37	0.221
Low density-lipoprotein cholesterol (mg/dl)	69 ± 7	72 ± 8	1.000
High density-lipoprotein cholesterol (mg/dl)	53 ± 9	63 ± 8	0.862
Triglyceride (mg/dl)	86 ± 19	127 ± 18	0.206
Creatinine (mg/dl)	0.45 ± 0.05	0.54 ± 0.05	0.264
eGFR (ml/min/1.73 m^2^)	136.8 ± 17.1	124.9 ± 19.8	0.214
FT4 (ng/ml)	4.95 ± 1.86	4.13 ± 2.08	0.228
FT3 (pg/ml)	16.34 ± 8.78	14.27 ± 8.29	0.649
TRAb (IU/l)	58.6 ± 102.8	24.1 ± 36.6	0.371
s(P)RR (ng/ml)	29.5 ± 5.1	25.0 ± 6.50	0.030

NR, non-responder; R, responder; eGFR, estimated glomerular filtration rate; FT4, free thyroxine; FT3, free triiodothyronine; TRAb, anti-TSH receptor antibody; s(P)RR, soluble (pro)renin receptor;

### Correlation between background factors and serum s(P)RR levels in GD patients

Results of single correlation analyses between background factors and serum s(P)RR levels in patients with GD are shown in Table 4. In GD patients, serum levels of triglyceride (TG) were negatively correlated with serum s(P)RR levels and those of FT4 were significantly positively correlated with serum s(P)RR levels. Serum TRAb values were not related with serum s(P)RR levels. Multiple regression analyses testing serum levels of TG and FT4 which showed single correlations with serum s(P)RR levels as independent variables revealed that TG but not FT4 levels were significantly negatively correlated with serum s(P)RR levels (Table 5).

**Table 4:** Single correlation analyses with serum s(P)RR levels in patients with Graves’ disease.

	R	p
Age	0.109	0.568
Body mass index	-0.057	0.760
Blood pressure		
Systolic blood pressure	0.106	0.590
Diastolic blood pressure	0.171	0.384
Blood tests		
Hemoglobin A1c	-0.117	0.613
Low density-lipoprotein cholesterol	-0.046	0.858
High density-lipoprotein cholesterol	-0.287	0.281
Triglyceride	-0.493	0.021
Creatinine	-0.192	0.370
eGFR	0.213	0.342
FT4	0.363	0.042
FT3	0.313	0.058
TRAb	0.164	0.369

s(P)RR, soluble (pro)renin receptor; eGFR, estimated glomerular filtration rate; FT4, free thyroxine; FT3, free triiodothyronine; TRAb, anti-TSH receptor antibody

**Table 5:** Multiple regression analyses with serum s(P)RR levels in patients with Graves’ disease.

	β	p
Triglyceride	-0.047	0.047
FT4	1.026	0.146
R^2^ = 0.280, P<0.001 for entire model		

s(P)RR, soluble (pro)renin receptor; FT4, free thyroxine

**Fig 1:**
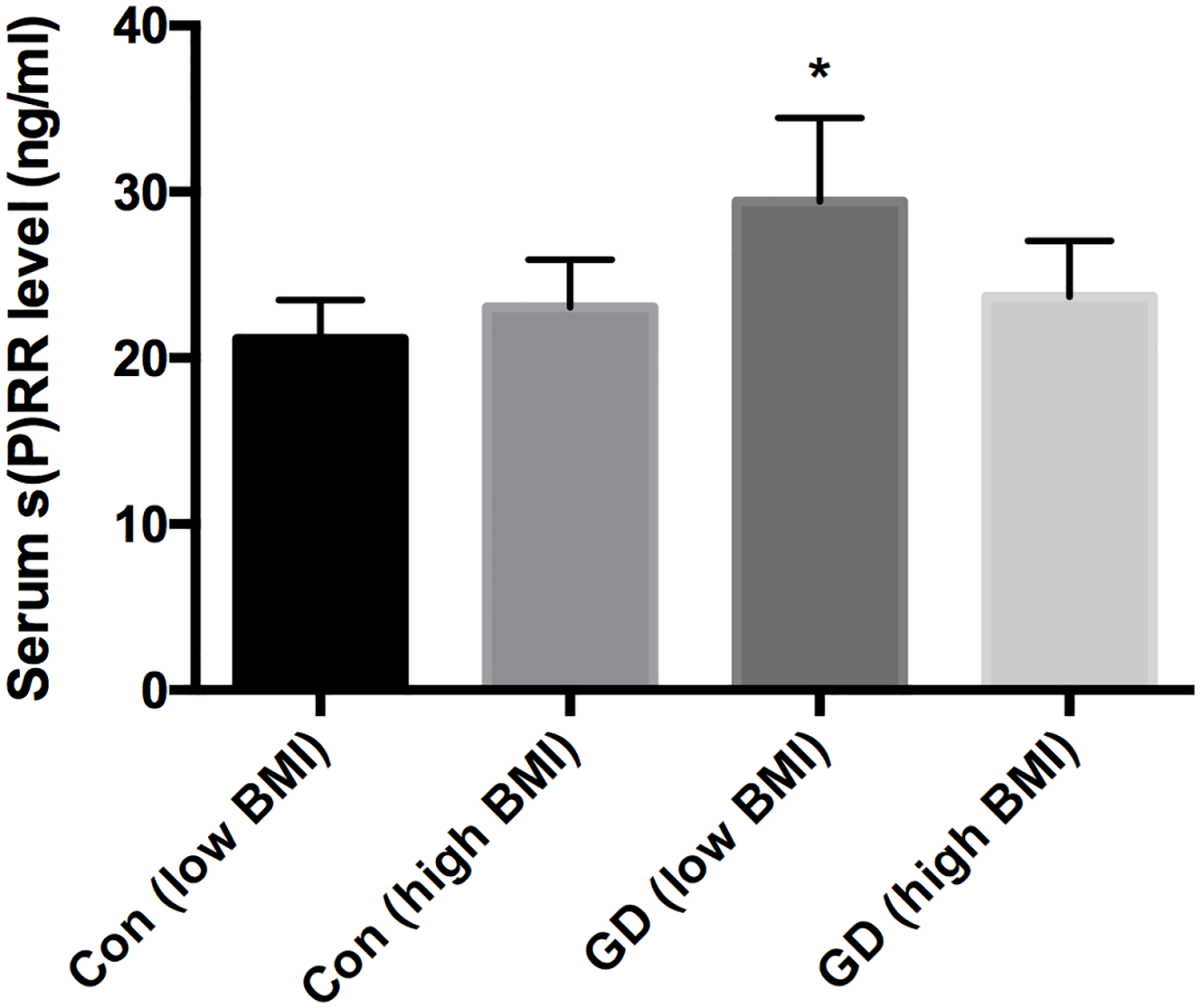
Comparisons of serum soluble (pro)receptor levels. *P < 0.0001 compared with GD (high BMI), Con (low BMI), and Con (high BMI). BMI, body mass index; Con, control; GD, Graves’ disease; low BMI, BMI < 22 kg/m^2^; high BMI, BMI ≥ 22 kg/m^2^; s(P)RR, soluble (pro)renin receptor.

When GD patients and control participants were divided into low BMI (< 22 kg/m^2^) and high BMI (≥ 22 kg/m^2^) groups, GD patients with low BMI showed higher levels of serum s(P)RR than both those with high BMI and control participants with low BMI and high BMI (p<0.0001 for each). There were no significant differences among control participants with low BMI and high BMI and GD patients with high BMI (Fig 1).

## Discussion

The present study demonstrated three major findings regarding the s(P)RR in GD patients. First, patients with hyperthyroidism due to GD showed increased serum s(P)RR levels which were significantly reduced after medical treatment for GD. Second, high serum s(P)RR levels were associated with resistance to antithyroid drug treatment, suggesting that serum s(P)RR concentration could be used as a biomarker to predict the outcome of antithyroid drug treatment in these patients. Third, serum TG levels were independently significantly correlated with serum s(P)RR levels in GD patients, and GD patients with low BMI showed higher levels of serum s(P)RR than those with high BMI, suggesting a possible relationship between low nutrient condition due to hyperthyroidism and increased s(P)RR levels in these patients.

### Serum s(P)RR levels in GD patients

It has been reported that thyroid hormones upregulate not only the endocrine RAS [23, 24] but also cardiac tissue RAS: thyroid hormones increase cardiac levels of renin and Ang II [25, 26] and Ang type 1 and type 2 receptors [23, 27]. The fact that thyroid hormones affect tissue RAS predisposed us to investigate the levels of serum s(P)RR as a possible marker for the tissue RAS status in patients with GD. Patients with hyperthyroidism due to GD showed increased serum s(P)RR levels (Table 1), which were significantly reduced after medical treatment for GD. This finding is interesting because it may suggest that activation of tissue RAS might be via increased (P)RR. However, this remains speculative and further studies are needed to investigate the roles of (P)RR in the alteration of tissue RAS in condition of thyroid hormone excess.

### Possible mechanisms for the relationships between serum s(P)RR concentration and efficacy of antithyroid treatment in GD patients

Intriguingly, in this study we found that GD patients with high serum s(P)RR levels show treatment resistance against antithyroid drugs (Table 2), while there were no significant differences in thyroid functions between NR and R groups (Table 3). These data suggest that treatment-resistance may not be just due to severe hyperthyroidism in the NR group and that serum s(P)RR concentration could be used as a biomarker to predict the outcome of antithyroid drug treatment. The reason for the latter, however, is unclear and needs to be discussed. (P)RR is known as a protein stabilizer which protects pyruvate dehydrogenase (PDH) complex from phosphorylation, contributing to the aerobic glucose metabolism [20]. Antithyroid drugs suppress the production of thyroid hormones by inhibiting thyroperoxidase. Therefore, the overexpression of (P)RR reflected by high serum s(P)RR levels may imply activation of tricarboxycylic acid cycle causing activation of peroxidase that is too active to be successfully inhibited by antithyroid drugs of clinical doses. However, this presumption needs to be tested in further studies.

### Relationship between serum levels of TG and s(P)RR

We have previously reported positive relationships between serum levels of TG and s(P)RR in patients with EH [8] and in hemodialysis patients [9], suggesting the presence of interactions between lipid metabolism dysfunction and serum s(P)RR levels. Although the reason for these associations is unknown, one may consider that patients with high TG levels may have increased expression of (P)RR in the hypertrophied adipose tissue to cause an elevation in serum s(P)RR levels. However, in this study, patients with GD showed conflicting data related to these associations. GD patients showed significant “negative” relationship between serum levels of TG and s(P)RR (Table 3). Furthermore, in our sub-analysis, GD patients with low BMI showed higher levels of serum s(P)RR than those with high BMI and control participants with low BMI and high BMI, and there were no significant differences among GD patients with high BMI and control participants with low BMI and high BMI (Table 5), suggesting it is unlikely that increased metabolic rate in Graves’ disease is the cause of increased s(P)RR.

These data raise the possibility that activated metabolism caused by hyperthyroidism is affecting this association. There may be a possible mechanism to overcome the low nutrient condition due to increased metabolism in GD. We have reported that the total ablation of (P)RR in vivo caused impairment of V-ATPase function and compromised intravascular acidification, which leads to the dysfunction of the autophagy system [4] [28]. Autophagy is triggered to compensate nutrient starvation [29]. It could be possible that (P)RR expression is increased to stimulate intracellular energy production via activating autophagy function. We speculate that (P)RR expression levels, and as a result serum s(P)RR levels, are elevated to compensate intracellular starvation in patients with GD with low BMI. We currently plan in vivo and in vitro experiments to examine whether low nutrient condition enhances expressions of (P)RR and s(P)RR, and the precise mechanisms underlying this phenomenon.

## Limitations

There are several limitations that should be acknowledged in the present study. First, this study involved a relatively small number of patients. Second, the origin of the increased s(P)RR in serum could not be determined from this study. Third, we could not directly investigate whether expression of (P)RR and other RAS components were increased in the tissues of the participants. Finally, the regulatory factors of serum s(P)RR levels were not determined. Further investigations to address these issues are required in larger-scale studies.

## Conclusions

Our data showed that patients with hyperthyroidism due to GD revealed increased serum s(P)RR levels which were significantly reduced after medical treatment for GD. High serum s(P)RR levels were associated with resistance to antithyroid drug treatment, suggesting the efficacy of serum s(P)RR levels as a biomarker to predict the outcome of antithyroid drug treatment in these patients. GD patients with low BMI showed higher levels of serum s(P)RR than those with high BMI and control subjects. In addition, in GD patients, TG levels but not thyroid hormone levels were independently significantly correlated with serum s(P)RR levels. Collectively, these data suggested a possible relationship between low nutrient condition due to hyperthyroidism and increased s(P)RR levels in GD patients. It could be possible that s(P)RR levels may be elevated in the process of stimulating intracellular energy production to overcome low nutrient condition due to hyperthyroidism especially in patients with low BMI, and the resultant increase in (P)RR expression may activate tissue RAS to cause organ damages. Future investigations are required to test this presumption and significance of interventions to modulate (P)RR expression in reducing organ damages in these patients.
